# Time Course and Role of Exercise-Induced Cytokines in Muscle Damage and Repair After a Marathon Race

**DOI:** 10.3389/fphys.2021.752144

**Published:** 2021-10-15

**Authors:** Cesar Augustus Zocoler de Sousa, Ana Paula Renno Sierra, Bryan Steve Martínez Galán, Jaqueline Fernanda de Sousa Maciel, Richelieau Manoel, Hermes Vieira Barbeiro, Heraldo Possolo de Souza, Maria Fernanda Cury-Boaventura

**Affiliations:** ^1^Interdisciplinary Post-graduate Program in Health Sciences, Institute of Physical Activity and Sports Sciences, Cruzeiro Do Sul University, São Paulo, Brazil; ^2^School of Physical Education and Sport, University of São Paulo, São Paulo, Brazil; ^3^Emergency Medicine Department, LIM-51, University of São Paulo, São Paulo, Brazil

**Keywords:** muscle damage, myocardial damage, inflammation, endurance exercise, myokines, muscle repair

## Abstract

Endurance exercise induces an increase in the expression of exercise-induced peptides that participate in the repair and regeneration of skeletal muscles. The present study aimed to evaluate the time course and role of exercise-induced cytokines in muscle damage and repair after a marathon race. Fifty-seven Brazilian male amateur marathon finishers, aged 30–55 years, participated in this study. The blood samples were collected 24 h before, immediately after, and 24 and 72 h after the São Paulo International Marathon. The leukogram and muscle damage markers were analyzed using routine automated methodology in the clinical laboratory. The plasma levels of the exercise-induced cytokines were determined using the Human Magnetic Bead Panel or enzyme-linked immunosorbent assays [decorin and growth differentiation factor 15 (GDF-15)]. A muscle damage was characterized by an increase in plasma myocellular proteins and immune changes (leukocytosis and neutrophilia). Running the marathon increased interleukin (IL)-6 (4-fold), IL-8 (1.5-fold), monocyte chemoattractant protein-1 (2.4-fold), tumor necrosis factor alpha (TNF-α) (1.5-fold), IL-10 (11-fold), decorin (1.9-fold), GDF-15 (1.8-fold), brain-derived neurotrophic factor (BDNF) (2.7-fold), follistatin (2-fold), and fibroblast growth factor (FGF-21) (3.4-fold) plasma levels. We also observed a reduction in musclin, myostatin, IL-15, and apelin levels immediately after the race (by 22–36%), 24 h (by 26–52%), and 72 h after the race (by 25–53%). The changes in BDNF levels were negatively correlated with the variations in troponin levels (*r* = −0.36). The variations in IL-6 concentrations were correlated with the changes in follistatin (r = 0.33) and FGF-21 (*r* = 0.31) levels after the race and with myostatin and irisin levels 72 h after the race. The changes in IL-8 and IL-10 levels had positive correlation with variation in musclin (*p* < 0.05). Regeneration of exercise-induced muscle damage involves the participation of classical inflammatory mediators, as well as GDF-15, BDNF, follistatin, decorin, and FGF-21, whose functions include myogenesis, mytophagia, satellite cell activation, and downregulation of protein degradation. The skeletal muscle damage markers were not associated to myokines response. However, BDNF had a negative correlation with a myocardial damage marker. The classical anti-inflammatory mediators (IL-10, IL-8, and IL-6) induced by exercise are associated to myokines response immediately after the race and in the recovery period and may affect the dynamics of muscle tissue repair.

## Introduction

The mechanical and metabolic stress (mitochondrial dysfunction and challenge by energy ADP/ATP ratio), induced by the repetitive contractions of muscle fibers, causes a disruption of the sarcolemma and extracellular matrix, swelling of mitochondria, dilation of the transverse tubule system, and fragmentation of the sarcoplasmic reticulum, thereby promoting the increased permeability of the membrane and efflux of myocellular proteins into the blood circulation (Peake et al., 2017; Hody et al., 2019). The loss of calcium homeostasis, oxidative stress, increased calpain activity, and inflammation contribute to muscle injury (Hody et al., 2019). An increase in the levels of systemic inflammatory mediators, such as interleukin (IL)-1 beta, IL-6, IL-8, IL-1ra, and IL-10, is induced during the muscle damage due to endurance exercise. However, the cellular sources of these mediators, such as endothelial cells, pericytes, fibroblasts, neutrophils and monocytes/macrophages, or skeletal muscle cells, utilize the inflammatory mediators in the process of muscle repair and regeneration (Peake et al., 2015).

In addition to the classical inflammatory mediators, the changes have been observed in more than 650 myokines in response to muscle contraction and differentiation, with autocrine, paracrine, and endocrine actions (Whitham and Febbraio, 2016; Safdar and Tarnopolsky, 2018; Piccirillo, 2019; Bay and Pedersen, 2020; Laurens et al., 2020). Despite the identification of hundreds of myokines, biological function has only been described in a small portion of myokines. Many of these myokines have paracrine biological functions in muscle protein synthesis or degradation, proliferation and differentiation of myoblasts, activation of satellite cells, organization and remodeling of the extracellular matrix, as well as modulation of muscle wasting, repair, and regeneration (Hoffmann and Weigert, 2017; Lee and Jun, 2019; Laurens et al., 2020). The cellular sources of myokines include myocytes, satellite cells, endothelial cells, residential macrophages, and fibroblasts (Hoffman and Weigert, 2017). The myokines primarily studied after aerobic exercise include myostatin, IL-6, irisin, IL-15, growth differentiation factor 15 (GDF-15), brain-derived neurotrophic factor (BDNF), fibroblast growth factor (FGF)-21, apelin, angiopoietin-like protein 4, and decorin (Safdar and Tarnopolsky, 2018; Piccirillo, 2019; Bay and Pedersen, 2020; Laurens et al., 2020). The levels of systemic hepatokines, such as follistatin, FGF-21, and angiopoietin-like protein 4, have also been studied during and after the acute exercise (Gonzalez-Gil and Elizondo-Montemayor, 2020). The release of exercise-induced peptides is dependent on the type of exercise and training protocol (Piccirillo, 2019; Domin et al., 2021).

Understanding the time course of a variety of different exercise-induced peptides can help highlight the circulatory markers that participate in the different phases of the muscle damage and repair, such as pro and anti-inflammatory phase, muscle and connective tissue remodeling as well as synthesis and protein degradation after long-distance exercise. The present study is the first study to determine and correlate a large number of muscle damage markers and inflammatory and tissue repair mediators in long-distance runners. Our hypothesis is that the extent of muscle damage may influence the inflammatory response, which in turn may affects the dynamics of muscle tissue repair during the recovery period.

The present study aimed to evaluate the time course and relationship of exercise-induced cytokines and muscle damage markers after a marathon race. An understanding of the course of the release of exercise-induced peptides on circulation and their association during muscle damage and repair may contribute to elucidate the endurance exercise muscle adaptations and may yield potential molecular therapeutic targets to treat the myopathies that involve mitochondrial dysfunctions.

## Materials and Methods

### Subjects

Fifty-seven amateur Brazilian male marathon finishers, aged 30–55 years, participated in this study. The recruitment of the volunteers was performed by the São Paulo International Marathon Organization (2017, Yescom, BRA) by mailing and the volunteers filled a form containing personal data, such as age, e-mail, address, and phone number. The researchers contacted the volunteers by phone to inform the more details of the collection steps, such as four blood collection and a cardiopulmonary exercise test to confirm interest and availability of a runner to participate in the research. Then, the runners were randomized after training and medical history. The exclusion criteria included the use of medication for cardiac, metabolic, pulmonary, or kidney injury, use of alcohol or any kind of drugs and pathologies, such as systemic arterial hypertension, liver, kidney, metabolic, inflammatory, or neoplastic diseases. The inclusion criteria were having already participated in one or more marathon or half marathon and being training for more than 30 km per week. The subjects were informed of the experimental procedures and possible risks and signed a term of informed consent approved by the Ethics Committee of Dante Pazzanese Institute of Cardiology, Brazil (Permit Number: 979/2010), in accordance with the Declaration of Helsinki. A cardiopulmonary exercise test (CPET) was performed 3–21 days before the marathon race using a treadmill protocol (TEB Apex 200, TEB, São Paulo, Brazil, speed 0–24 km/h, grade 0–35%). The test was performed in 1% fixed slope and speed began with 8 km/h increasing 1 km/h per minute until the maximal exhaustion of the runner. An expired gas analysis was performed in a breath-by-breath system (Quark CPET, Cosmed, Rome, Italy). During the test, the runners were monitored with a standard 12-lead electrocardiogram (4 limb and 6 thorax electrodes, ECG, TEB, São Paulo, Brazil) to check for the possible cardiac changes during exertion. All the runners recruited completed the International Marathon of São Paulo 2017.

The measurements of total body mass (kg), height (cm), and Body Mass Index (BMI, kg/m^2^) were conducted 1-day before the marathon race at Cruzeiro do Sul University, São Paulo, Brazil, according to the International Society for the Advancement of Kinanthropometry and expressed as the mean ± SEM. The body composition (percentage of fat mass and free fat mass) was assessed by the bioimpedance analysis (Biodynamics Corporation, Seattle, WA, USA, 310e) with the runners fasting for at least 6 h 1-day before the marathon.

The blood samples (30 ml) were collected in vacuum tubes containing an anticoagulant [0.004% ethylenediaminetetraacetic acid [EDTA]]. Before, 24 h after, and 72 h after blood samples were collected at the Institute of Physical Activity and Sports Science (Cruzeiro do Sul University) and 20 ml forwarded to the clinical laboratory (Associação Fundo de Incentivo à Pesquisa, AFIP) for immediate skeletal and cardiac muscle damage markers and leukogram analyses and 10 ml were immediately centrifugated for plasma collection, and storage at −80°C, for the subsequent exercise-induced cytokine analyses. The blood samples immediately after the race were collected from a research area located close to the finish line and 20 ml forwarded to the clinical laboratory (Associação Fundo de Incentivo à Pesquisa, AFIP) and 10 ml to the Institute of Physical Activity and Sports Science for the same analyses.

### Marathon Race

The São Paulo International Marathon (2017) started at 07:30 a.m. on April 9. Fluid ingestion was allowed *ad libitum* during the race. Water was available every 2–3 km on the running course; sports drinks were available at 12, 21.7, 33, and 42 km; and a potato was available at 28.8 km. The weather parameters between 07:00 a.m. and 02:00 p.m. were as follows: average temperature, 19.8°C; average relative humidity, 72.8% (the National Institute of Meteorology, Ministry of Agriculture, Livestock, and Supply, Brazil).

### Muscle Damage Markers and Leukogram

The muscle damage markers were evaluated using a routine automated methodology in the Clinical Laboratory (Associação Fundo de Incentivo à Pesquisa, AFIP), immediately after the blood collection (20 ml). The creatine kinase (CK) and lactate dehydrogenase (LDH) activities were determined *via* a kinetic assay; troponin (I), N-terminal pro B-type natriuretic peptide (NT-proBNP), and myoglobin levels, and creatine kinase MB (CKMB) activity, were evaluated using a chemiluminescence assay. Leukogram was constructed *via* measurement using the cytochemical/isovolumetric method, and the C-reactive protein (CRP) levels were quantified *via* immunoturbidimetry assay.

### Determination of Cytokines Induced by Exercise

The plasma levels of interleukin (IL)-1ra, IL-4, IL-10, tumor necrosis factor alpha (TNF-α), vascular endothelial growth factor (VEGF), fibroblast growth factor-2 (FGF-2), interferon-gamma (IFN-gamma), macrophage inflammatory protein-1 (MIP-1), and monocyte chemoattractant protein-1 (MCP-1) were determined using the MILLIPLEX® Human Cytokine/Chemokine Magnetic Bead Panel (HCYTOMAG-60K, EMD Millipore Corporation, MA, USA). The plasma levels of apelin, irisin, BDNF, myostatin, musclin, follistatin, IL-6, IL-15, and FGF-21 were determined using the MILLIPLEX® Human Myokine Magnetic Bead Panel (HCYTOMAG-56K, EMD Millipore Corporation, MA, USA), according to the instructions of the manufacturer. The decorin and growth differentiation factor 15 (GDF-15) levels were determined ***via*** enzyme-linked immunosorbent assays (Duoset-ELISA, R&D Systems, USA).

The intra assay precision (mean of the percentage of coefficient variation) described by the protocols of the manufacturer were <3% to IL-1ra, IL-6, IL-10, IL-15, FGF-2, IFN-gamma, MIP-1 and were <10% to TNF-α, VEGF, MCP-1, apelin, irisin, BDNF, myostatin, musclin, follistatin, FGF-21, GDF-15, and decorin.

### Statistical Analyses

The statistical analyses were performed using GraphPad Prism (GraphPad Prism version 9, San Diego, CA, USA). The normality of the data distribution was determined using the Kolmogorov–Smirnov test and the normality was rejected. The general and training characteristics were described as the mean ± SEM. The statistical analyses were evaluated using one-way repeated measures ANOVA test, Geisser–Greenhouse correction for sphericity and Holm–Sídák post-test for multiple comparisons between before vs. immediately after, 24 and 72 h after the race. The non-parametric Spearman correlations were determined between the changes in muscle damage marker levels and general characteristic and training characteristic data; between the leukogram and general characteristic and training characteristic data; between the cytokine levels and general characteristic and training characteristic data (before, immediately after the race, or 24 h after the race); and between the changes in cytokine and muscle damage marker levels. The changes (after the race–before the race) in the levels of troponin, proBNP, LDH, leukogram, IL-6, IL-8, MCP-1, TNF-alpha, IL-10, apelin, decorin, GDF-15, BDNF, follistatin, and FGF-21 were calculated. The variations (24 h after the race–before the race) in CK and CKMB activities, and CRP level were calculated and the changes (72 h after the race–before the race) in IL-15, irisin, myostatin, apelin, and IL-6 levels were calculated. The statistical significance was assumed at *p* < 0.05. In the graph, the absolute values presented comprise the minimum, maximum, median, and outliers, with respect to the 40–57 runners. To perform one-way repeated measures ANOVA test, four runners were excluded from the analysis of leukogram, CRP, and muscle damage markers for not having completed the four blood collections and 10–13 runners were excluded from the analysis of cytokines with no detectable value before, after, 24 h, or 72 h after the race.

## Results

### General Characteristics

The general and training characteristics of amateur marathon runners are summarized as follows: age, 41.1 ± 0.9 years; weight, 74.8 ± 2.7 kg; height, 1.73 ± 0.01 m; BMI, 24.3 ± 0.5 kg/m^2^; percentage of fat mass, 21.8 ± 0.6%; free fat mass, 57.7 ± 0.9 kg; race time 248.9 ± 5.8 min, training experience, 7.4 ± 0.7 years; time in 10 km race, 46.5 ± 0.82 min; frequency of training, 4.2 ± 0.15 times/week; and training volume, 55.1 ± 4.9 km/week. The CPET parameters are summarized as follows: maximum speed of runners was 18.9 ± 0.3 km/h; time of exhaustion 11.5 ± 0.3 min; anaerobic threshold oxygen consumption (VO_2_ AT), 34.5 ± 1.0 ml/kg/min; respiratory compensation point oxygen consumption (VO_2_ RCP), 53.9 ± 1.2 ml/kg/min; and peak oxygen consumption (VO_2_ peak), 56.0 ± 1.3 ml/kg/min.

### Muscle Damage Markers and Leukogram

**Figure 1:**
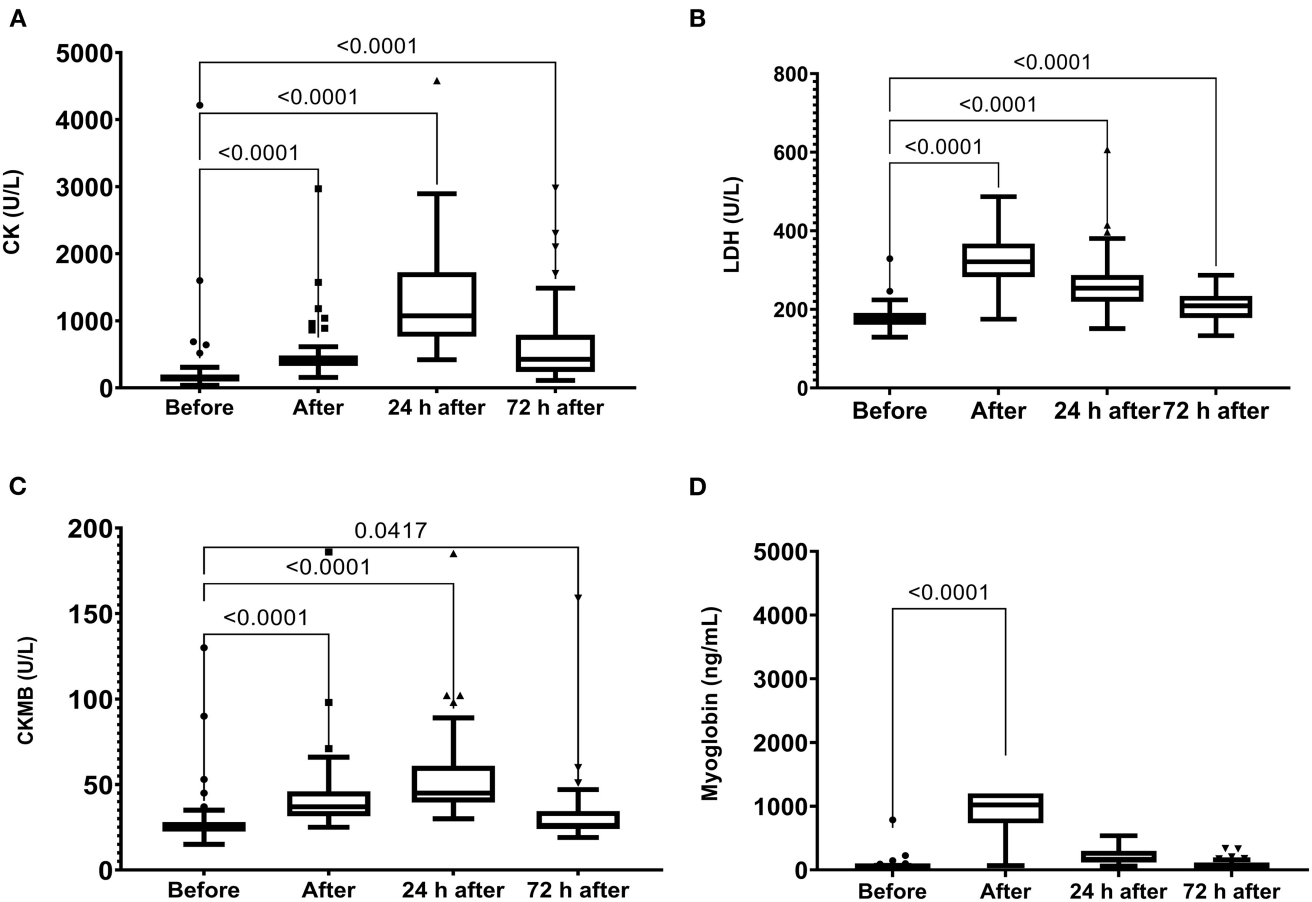
Creatine kinase (CK) **(A)**, lactate dehydrogenase (LDH) **(B)**, Creatine kinase MB (CKMB) **(C)** activities and myoglobin **(D)** plasma levels immediately after, 24 h after, and 72 h after the race. Creatine Kinase, CK, Lactate Dehydrogenase, LDH. The values are presented as minimum, maximum, median, and outliers of 53 runners (Box plot, Tukey).

**Figure 2:**
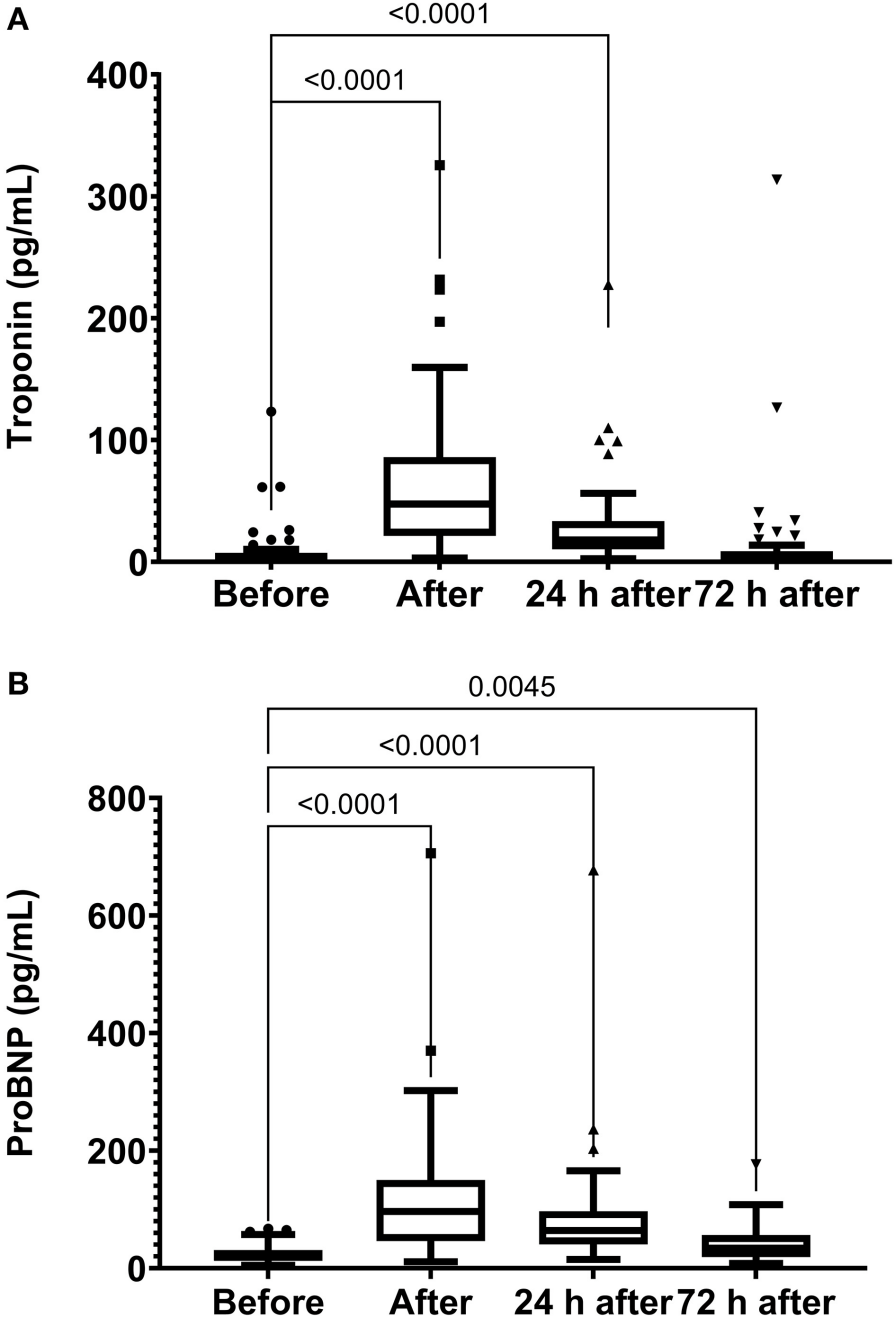
Troponin **(A)** and NT-proBNP **(B)** levels immediately after, 24 h after, and 72 h after the race. N-Terminal pro B-type natriuretic peptide, proBNP. The values are presented as minimum, maximum, median, and outliers of 53 runners (Box plot, Tukey).

**Figure 3:**
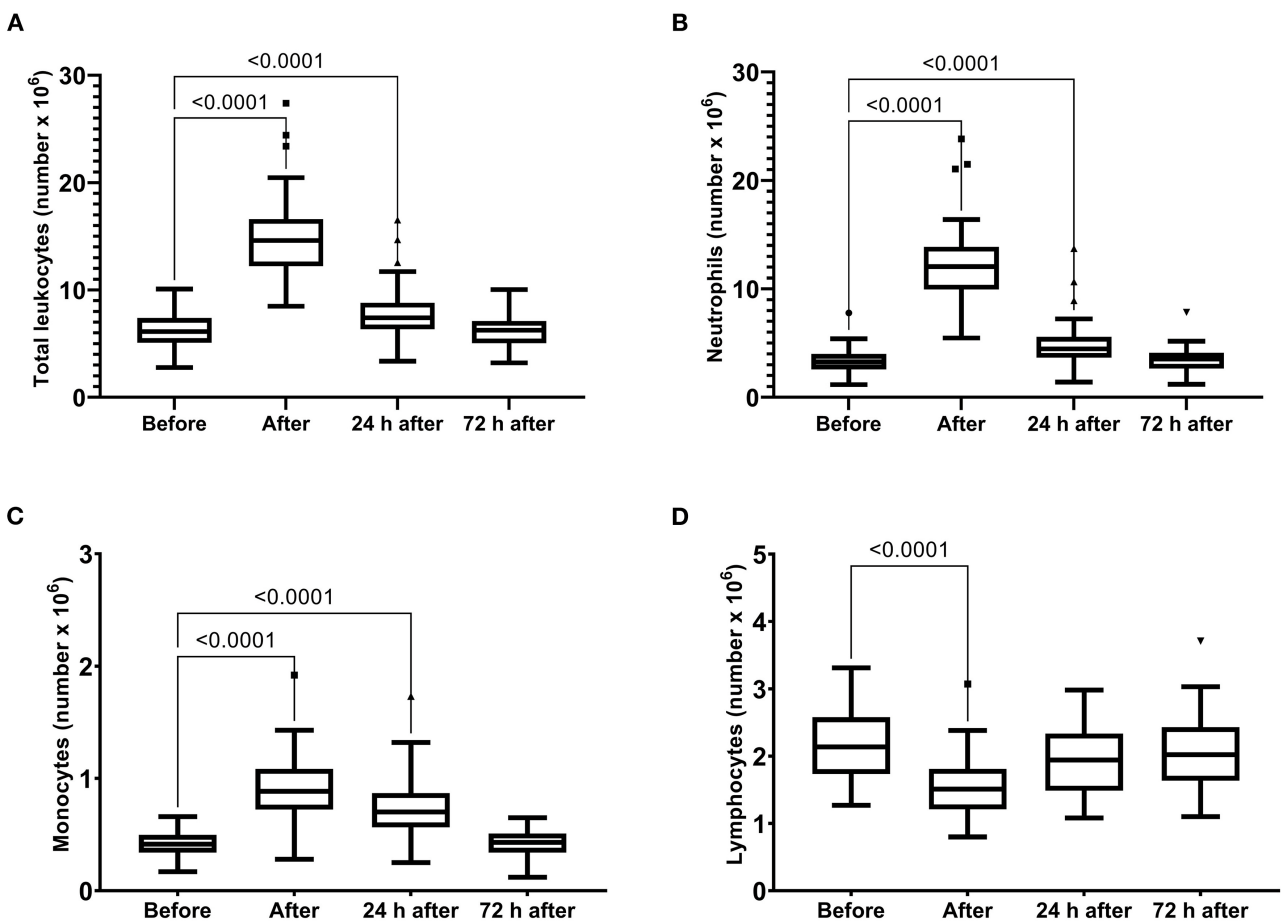
Number of total leukocytes **(A)**, neutrophils **(B)**, monocytes **(C)** and lymphocytes **(D)** immediately after, 24 h after, and 72 h after the race. The values are presented as minimum, maximum, median, and outliers of 53 runners (Box plot, Tukey).

The skeletal muscle damage was characterized by an increase in CK, LDH, and CKMB activity and myoglobin concentration after the race until 72 h after the race (Figure 1). In addition, we observed myocardial damage by the elevation of troponin and proBNP levels after the race (Figure 2). The total leukocytes, neutrophils, and monocytes were elevated after the race and 24 h after the race (Figures 3A–C); level of lymphocytes decreased after the race (Figure 3D), demonstrating immune changes after the race. We also observed an increase in the CRP levels, 24 h after the race, until 72 h after the race (from 0.83 ± 0.31 to 2.2 ± 0.2 to 0.9 ± 0.04 mg/dl).

The variations in the total number of leukocytes, neutrophils, and monocytes were correlated with the variations in CKMB and LDH activities and troponin and myoglobin levels (Table 1). The changes in CRP were correlated with the variations in troponin (*p* = 0.043, *r* = −0.27) and NT-proBNP levels (*p* = 0.037, *r* = −0.38), indicating a suitable marker of myocardial damage after the race.

**Table 1:** Correlation of changes in the number of leukocytes and changes in the muscle damage markers.

	**Δ** **Number of leukocytes**		**Δ** **Number of neutrophils**		**Δ** **Number of monocytes**	
	***p***	***r***	***p***	***r***	***p***	***r***
Δ CKMB	0.0006	0.44	0.0001	0.48	0.037	0.27
Δ Troponin	0.013	0.36	0.019	0.31	0.0014	0.41
Δ Myoglobin	0.032	0.28	0.0011	0.33	NS	NS
Δ LDH	0.0004	0.45	<0.0001	0.5	NS	NS

*CKMB, Creatine kinase MB; LDH, lactate dehydrogenase, NS, not significantly; Δ, Delta of variation*.

### Time Course of Exercise-Induced Cytokines

**Figure 4:**
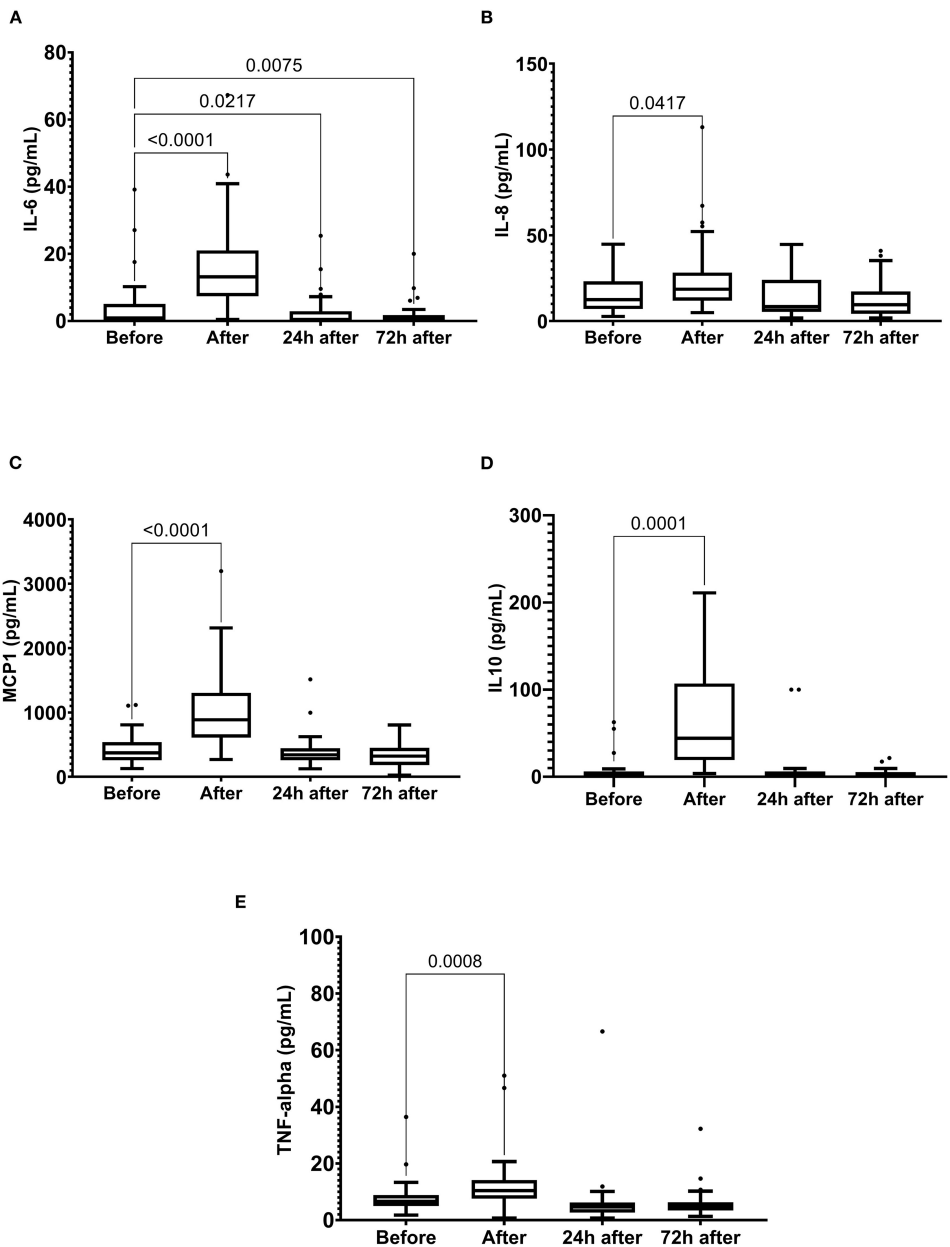
The plasma levels of interleukin (IL)-6 **(A)**, IL-8 **(B)**, monocyte chemoattractant protein-1 (MCP1) **(C)**, IL-10 **(D)**, and tumor necrosis factor alpha (TNF-α) **(E)** immediately after, 24 h after, and 72 h after the race. The values are presented as minimum, maximum, median, and outliers of 40 runners (IL-6) or 43 runners (MCP1, TNF-alpha, and IL-10) (Box plot, Tukey).

**Figure 5:**
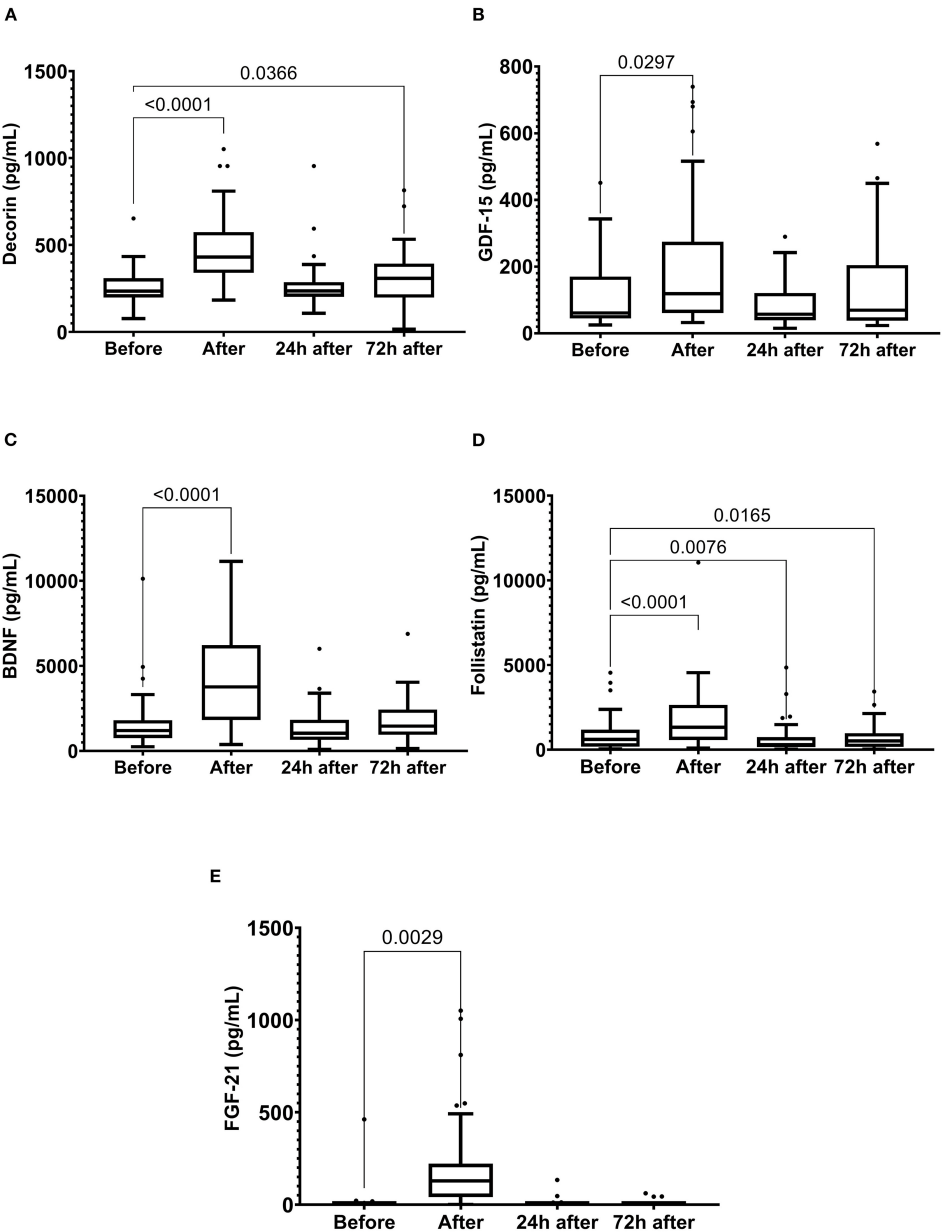
The plasma levels of decorin **(A)**, growth differentiation factor 15 (GDF-15) **(B)**, brain-derived neurotrophic factor (BDNF) **(C)**, follistatin **(D)** fibroblast growth factor 21 (FGF-21) **(E)** immediately after, 24 h after, and 72 h after the race. The values are presented as minimum, maximum, median, and outliers of 48 runners (decorin, GDF-15, BDNF, and Follistatin) or 40 runners (FGF-21) (Box plot, Tukey).

Running the marathon led to the elevations in IL-6, IL-8, MCP-1, IL-10, and TNF-α plasma levels (Figure 4). IL-6 concentration reduced 24 and 72 h after the race (Figure 4A) and IL-8, MCP-1, TNF-α, and IL-10 plasma levels returned to the basal levels 24 h after the race (Figures 4B–E). The decorin, GDF-15, BDNF, follistatin, and FGF-21 levels were increased immediately after the race (Figure 5). Decorin elevated 72 h after the race (Figure 5A) and follistatin decreased 24 and 72 h after the race (Figure 5D). The GDF-15, BDNF, and FGF-21 levels returned to the basal levels 24 h after the race (Figures 5B,C,E).

**Figure 6:**
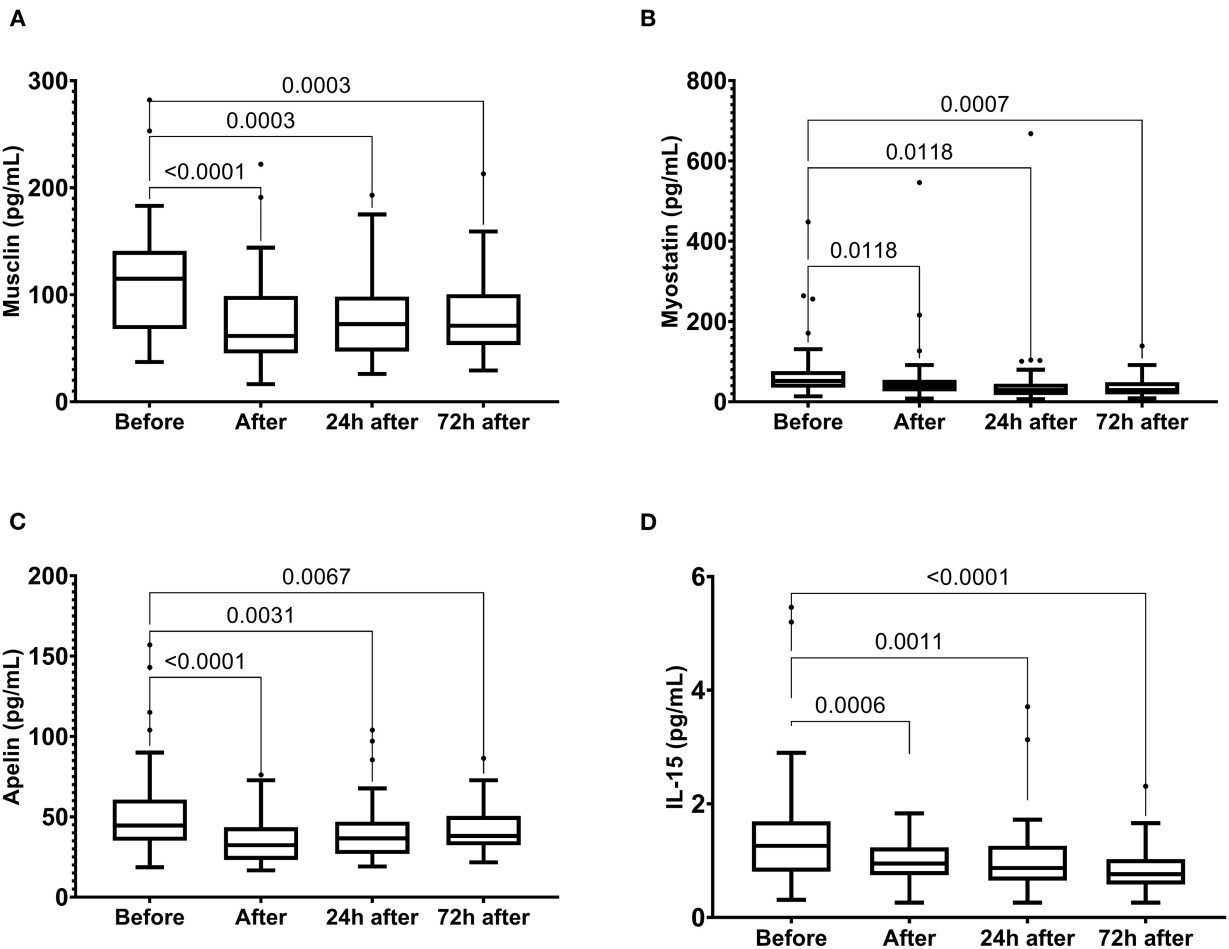
The plasma levels of musclin **(A)**, myostatin **(B)**, apelin **(C)** and IL-15 **(D)** immediately after, 24 h after, and 72 h after the race. The values are presented as minimum, maximum, median, and outliers of 48 runners (Box plot, Tukey).

In addition, we observed a reduction in musclin, myostatin, apelin, and IL-15 levels immediately after the race, which were maintained until 72 h after the race (Figure 6).

We did not observe the changes in plasma levels of VEGF, FGF-2, IFN- gamma, IL-4, IL-1ra, irisin, and MIP-1-alpha (data not shown).

### Correlation: General Characteristics

The changes in the CRP levels were correlated with body mass (*p* = 0.012, *r* = −0.33), BMI (*p* = 0.018, *r* = −0.31), and the percentage of fat mass (*p* = 0.0007, *r* = −0.43), and VO_2peak_ (0.005, *r* = −0.37). We observed no association between the changes in muscle damage markers and body composition (data not shown).

**Figure 7:**
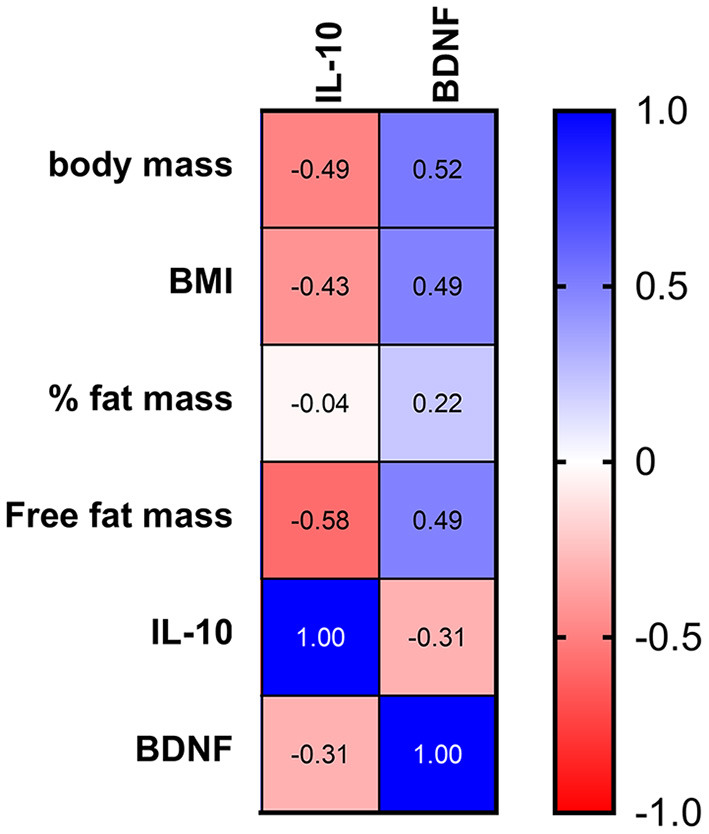
Correlation between the body composition and changes in IL-10 and BDNF after the race. The values are presented as Spearman's r of 43 runners for IL-10 and 38 runners for BDNF. Colormap with range between 1 and −1 and blue for the largest value and red for the smallest value.

The changes in the BDNF levels were positively correlated with body mass, BMI, percentage of fat mass, and free fat mass, while the variations in IL-10 levels exhibited a negative correlation with these body composition parameters (Figure 7), suggesting a greater anti-inflammatory response and impairment of the BDNF response in runners with lower free fat mass. In addition, the changes in myostatin exhibited a negative correlation with BMI (*p* = 0.24, *r* = −0.32).

The training experience was positively correlated with the changes in the total leukocyte and neutrophil counts and LDH activity (*p* < 0.05, *r* = 0.30). VO_2peak_ correlated with the variations in monocytes (*p* = 0.018, *r* = −0.31). The training experience was negatively correlated with the changes in those BDNF (*p* = 0.008, *r* = −0.38) and GDF-15 levels (*p* = 0.02, *r* = −0.31) and positively correlated with the IL-10 levels (*p* = 0.025, *r* = 0.35). The training experience exhibited a positive correlation with the absolute values of GDF-15, musclin, myostatin, and irisin levels before the race (*p* < 0.05, *r* = 0.30). The race time was negatively correlated with the IL-10, myostatin, and irisin levels (*p* < 0.05, *r* = −0.33).

### Correlations Between Exercise-Induced Cytokines Levels and Muscle Damage Markers

After the race, the changes in LDH activity correlated with the variations in those IL-10 (*p* = 0.010, *r* = 0.49) and TNF-α (*p* = 0.03, *r* = 0.33). The changes in CKMB activity also correlated with the variations in IL-10 levels (*p* = 0.010, *r* = 0.39). No association was found between the skeletal muscle damage markers and myokines analyzed in this study. Moreover, we observed a negative correlation between the changes in troponin and BDNF levels (*p* = 0.012, *r* = −0.37), indicating lower myocardial damage marker levels in the runners with greater BDNF response.

### Correlations Associated With Exercise-Induced Cytokine Levels

The changes in IL-6 concentration were correlated with the changes in follistatin (*p* = 0.009, *r* = 0.37) and FGF-21 levels (*p* = 0.007, *r* = 0.40). The changes in IL-8 and IL-10 levels had positive correlated with the variation in musclin (*p* = 0.013, *r* = 0.39 and *p* = 0.034, *r* = 0.34, respectively). The changes in follistatin concentration were positively correlated with the changes in BDNF (*p* < 0.0001, *r* = 0.55) and FGF-21 (*p* = 0.041, *r* = 0.31). The variations in musclin had a strong correlation with the variations in Apelin (*p* < 000.1, *r* = 0.70).

The changes in FGF-21 levels were negatively correlated with the variations in those of decorin (*p* = 0.039, *r* = −0.32) and apelin levels (*p* = 0.019, *r* = −0.35).

**Figure 8:**
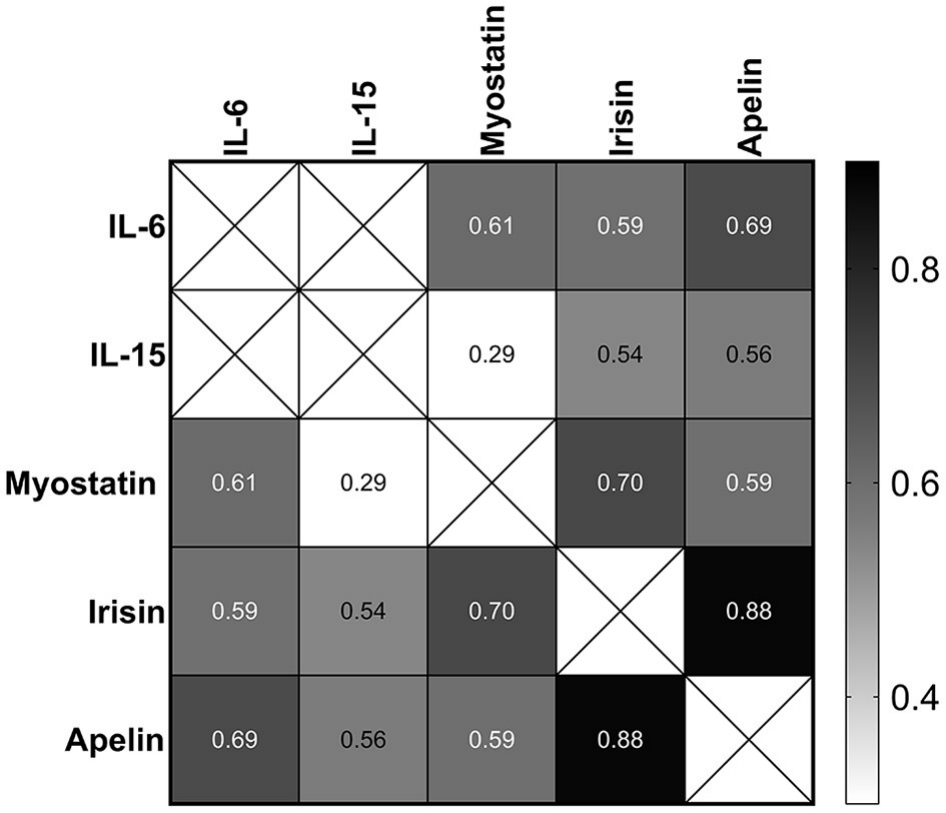
Correlation between the changes in IL-6, IL-15, myostatin, irisin, and apelin 72 h after the race. The values are presented as Spearman's r of 39 runners for irisin, 40 runners for apelin, and 43 runners for IL-6, IL-15, and myostatin. Colormap with range between 0.3 and 0.9 and black for the largest value and white for the smallest value.

In the recovery period, the changes in IL-6 (72 h after the race) were correlated with the changes in those myostatin, irisin, and apelin levels (*p* < 0.0001). The changes in IL-15 also were correlated with in those myostatin (0.038), irisin (*p* < 0.0001), and apelin levels (*p* < 0.0001). The changes in apelin and irisin also had strong correlation with the changes in myostatin (*p* < 0.0001) (Figure 8).

## Discussion

**Figure 9:**
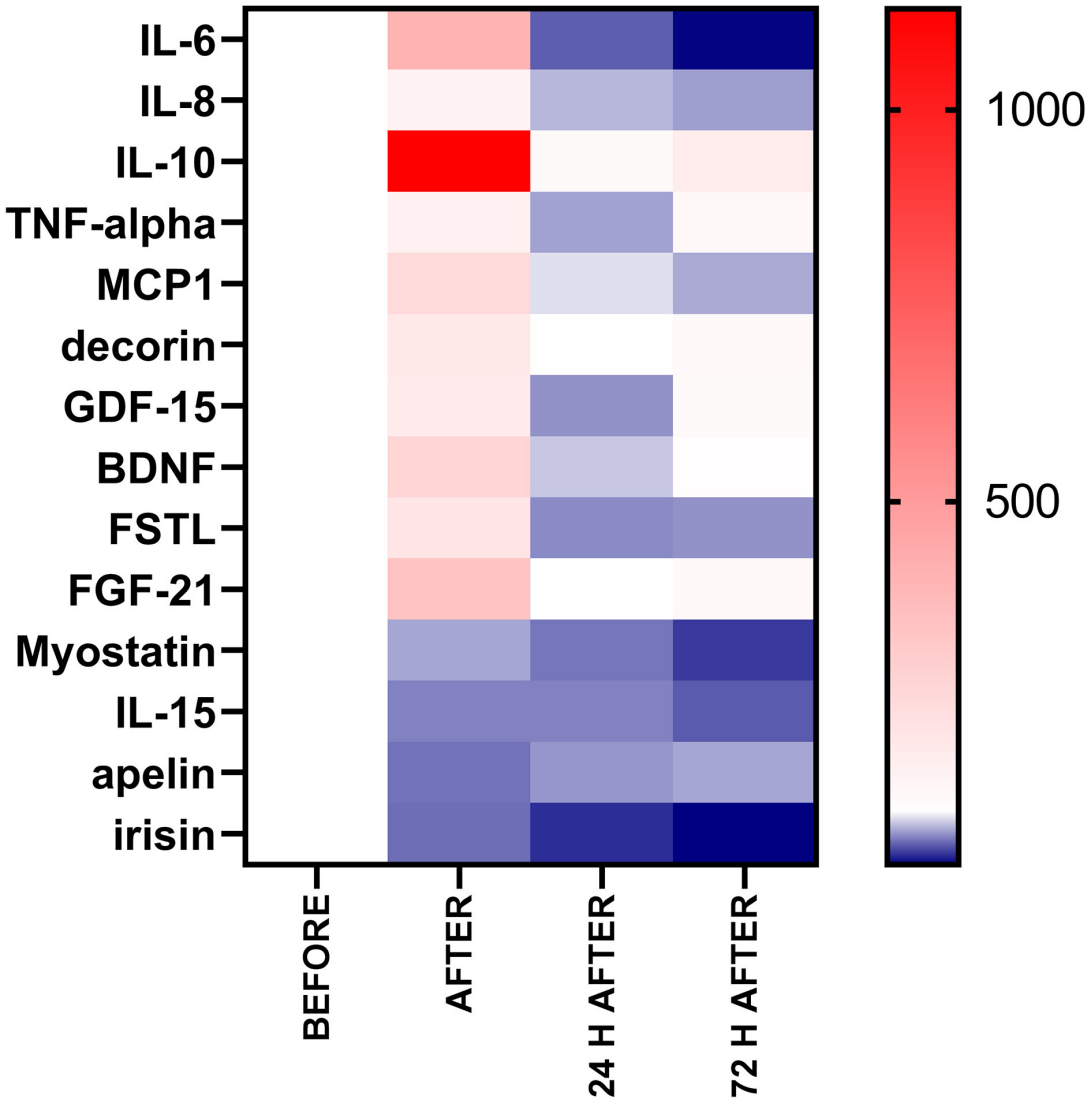
Percentage of baseline value of the exercise-induced cytokines after, 24 h after, and 72 h after. The values are presented as percentage of baseline value (before) compared with after, 24 h after, and 72 h after the race of 38–54 runners. Colormap with range between 1,129 and 37% and red for the largest value, white for the baseline (100%) and blue for the smallest value.

Immediately after the race, we observed leukocytosis, neutrophilia, and an increase in the muscle damage markers, IL-6, IL-8, IL-10, TNF-α, MIP-1, decorin, GDF-15, BDNF, follistatin, and FGF-21, owing to a reduction in the myostatin, musclin, IL-15 and apelin levels which were maintained reduced 72 h after the race (Figure 9). The muscle damage marker, plasmatic LDH activity, correlated with the mediators of inflammation (IL-10 and TNF-α) but not with myokines response. The classical anti-inflammatory mediators induced by exercise (IL-6 and IL-10) and IL-8 seem to be associated with myokines that affect the muscle repair. IL-10 and IL-8 response was associated with the musclin response, while IL-6 response was positively correlated with the FGF-21 and follistatin response after the race and with myostatin, apelin, and irisin response in the recovery period. Moreover, BDNF had a negative correlation with the troponin levels and further studies should be carried out to verify the role of BDNF levels in myocardial damage-induced by the race.

Leukocytosis after long-distance exercise is a result of hemodynamic and catecholamine induced demargination of vascular and pulmonary pools followed by a cortisol-induced release of neutrophils from the bone marrow. In the recovery period, these stress hormones also contribute to lymphocytopenia and both innate and acquire immune cell dysfunction (Nieman and Mitmesser, 2017; Jones and Davison, 2019; Suzuki et al., 2020). Muscle tissue infiltration of the innate immune cells is regulated by chemokines, such as IL-8 (Nieman and Mitmesser, 2017; Jones and Davison, 2019; Suzuki et al., 2020). The endurance exercise induces a pro inflammatory response mediated by the neutrophils and pro inflammatory macrophages, M1 in the early hours after exercise, follow an anti-inflammatory compensatory response mediated by M2 macrophages and T cells (Treg and CD8+) leading a period of immunosuppression called “open window” (Peake et al., 2017; Jones and Davison, 2019; Suzuki et al., 2020).

In this study, the levels of skeletal muscle damage markers were noted to be correlated with the immune changes and inflammatory mediators (IL-10 and TNF-α); however, no association was found between the variations in the levels of skeletal muscle damage markers and myokines. We suggest that regardless of the extent of skeletal muscle damage, other intrinsic muscle factors may modulate the release of myokines, as indicated by an association of free fat mass and/or training experience with the variations in BDNF, GDF-15, irisin, and myostatin levels. We observed an association between age and GDF-15, before and after the race, as proposed by Conte et al. (2020); however, we did not observe a correlation with the total number of leukocytes, neutrophils, and lymphocytes showed in the cyclists after exhaustive exercise (Conte et al., 2020). A GDF-15 is a stress-induced cytokine released from the TGF-β superfamily, in response to the mitochondrial stress and/or inflammatory stress. A GDF-15 has been suggested to induce reorganization and adaptation of systemic metabolism (Chung et al., 2017; Conte et al., 2020; Laurens et al., 2020). Although the contracting skeletal muscle releases GDF15, leading to an increase in the plasma levels, the source of systemic GDF-15 levels after acute exercise remains unclear (Kleinert et al., 2018; Conte et al., 2020).

IL-6, induced by exercise, has a well-known anti-inflammatory effect that modulates the release of IL-10 and IL-1ra. The other paracrine effects of IL-6 on the skeletal muscle include intramuscular lipolysis and improvement of insulin sensitivity and glucose intake (Carey et al., 2006; Wolsk et al., 2010; Laurens et al., 2020). Moreover, the previous studies have observed that an IL-6 induces the following: myogenic differentiation in the C2C12 myoblast cell line and primary human myoblasts, myotube protein synthesis in the C2C12 myoblast cell line, and murine and myoblast proliferation in the human satellite cell proliferation (Pedersen et al., 2001; Gao et al., 2017; Steyn et al., 2019). The studies in animals have elucidated the role of IL-6 in the activation of M2 macrophages, which promotes angiogenesis and damages tissue repair (Pilny et al., 2019), satellite cell proliferation, and myonuclear accretion (Serrano et al., 2008).

In this study, in response to the exercise, the IL-6 expression was positively correlated with that of FGF-21 and follistatin, which are considered hepatokines, rather than the myokines (Domin et al., 2021). Follistatin has myogenic properties that directly inhibit myostatin from binding to the activin IIb receptor and suppression of small mothers against decapentaplegic 3 (Smad3) phosphorylation, consequently increasing the protein synthesis by the mTOR/S6K/S6RP signaling cascade and contributing to skeletal muscle mass (Winbanks et al., 2012; Hoffmann and Weigert, 2017; Lee and Jun, 2019). In our study, the follistatin levels were correlated with those of BDNF, which are responsible for the activation and proliferation of satellite muscle cells after injury (Hoffmann and Weigert, 2017; Lee and Jun, 2019). BDNF had a negative correlation with a myocardial damage marker. A recent review highlighted that BDNF acts on myocardial tissue by decreasing the cardiomyocyte apoptosis and mitochondrial dysfunction and increasing angiogenesis, cardiomyocyte contraction, and calcium cycling *via* tropomyosin-related kinase receptor B (TrkB) signaling pathways (Hang et al., 2021).

Mitochondrial dysfunction and endoplasmic reticulum stress trigger the FGF-21 expression in the skeletal muscles (Tezze et al., 2019). FGF-21 modulates PI3K-AKT signaling, activates ATF4 in skeletal muscle, and is involved in the removal of damaged mitochondria *via* mitophagy and alteration of the type of muscle fiber; thus, it is involved in the regulation of both the muscle mass and function (Hoffmann and Weigert, 2017; Oost et al., 2019; Lee and Jun, 2019). In addition, FGF-21 seems to have metabolic effects similar to those of IL-6 in the skeletal muscle (Tanimura et al., 2016; Struik et al., 2019; Tezze et al., 2019). The FGF-21 response correlated negatively with the decorin and apelin response, indicating that better FGF-21 response may avoid decorin and apelin reduction after the race.

Decorin is a member of the small leucine-rich proteoglycan family of extracellular matrix proteins that interact with the collagen fibers. It has been reported to modulate the proliferation of human skeletal muscle cells and autophagy and the consequent promotion of muscle regeneration (Li et al., 2007); moreover, it directly inhibits myostatin by activating the SMAD-2/3 complex, thereby reducing the degradation of proteins in the skeletal muscle (El Shafey et al., 2016).

In the recovery period, IL-6 correlated with the myostatin, irisin, and apelin levels. Myostatin acts on the activin receptors (type I and II), promoting the phosphorylation and activation of SMAD proteins. SMAD-2 and SMAD-3 form a complex with SMAD-4, which induces the transcription of catabolic genes. Moreover, myostatin participates in the process of protein degradation through the ubiquitin-proteasome system and autophagy (Hoffmann and Weigert, 2017; Lee and Jun, 2019; Piccirillo, 2019).

The decrease in the levels of other well-known myokines that promote muscle regeneration and modulate autophagy, such as IL-15, apelin, and musclin, indicate that these molecules do not play decisive roles in the repair and regeneration after endurance exercise-mediated muscle damage. IL-15 is a myokine whose levels affect the differentiation of myoblasts, and this myokine modulates autophagy (Hoffmann and Weigert, 2017; Lee and Jun, 2019; Piccirillo, 2019; Trovato et al., 2019; Pesce et al., 2020); moreover, IL-15 is associated with the vascular smooth muscle cell proliferation, stem cell proliferation and differentiation, mitochondriogenesis, autophagy, and anti-inflammatory properties in skeletal muscle (Mughal and O'Rourke, 2018; Vinel et al., 2018).

The exercise-induced inflammatory mediators (IL-10 and IL-8) correlated with the changes in musclin which contains a region homologous to the members of the natriuretic peptide (NP) family. Musclin seems to promote the skeletal muscle oxidative capacity by mitochondrial biogenesis (Subbotina et al., 2015). In contrast to the studies in animals (Farrash et al., 2021), our data showed a decrease of musclin after acute exercise.

Daily dietary intake and food intake during the competition may affect the inflammatory response (Nieman and Mitmesser, 2017). A previous study of our group demonstrated the association of energy, electrolyte, and carbohydrate intakes and inflammatory response after endurance exercise (Passos et al., 2019). The limitation of this study was that it did not assess the role of food intake during the marathon race. Further studies are needed to evaluate the role of daily dietary intake, nutritional supplements, and food intake during the race on the exercise-induced cytokines.

Based on the results of the current study, we conclude that the endurance exercises, such as a marathon could alter the levels of inflammatory markers, and the interaction between the peptides shows that an increase in GDF-15, BDNF, follistatin, decorin, and FGF-21 expression, owing to the decrease in myostatin levels during the recovery period, appears to contribute to the muscle repair and regeneration after a long and high-intensity race. In addition, the regeneration of exercise-induced muscle damage involves the functioning of classical inflammatory mediators and myokines, whose functions include mediation of angiogenesis (IL-6), myogenesis (IL-6 and follistatin), mytophagia (FGF-21), and satellite cell activation (BDNF), in addition to the downregulation of protein (follistatin and decorin) degradative pathways. Additionally, further studies should be carried out to verify the role of BDNF levels in myocardial damage-induced by the race. Furthermore, IL-6 modulates the anti-inflammatory and metabolic responses, and it is associated with the levels of myokines (follistatin, FGF-21, myostatin, apelin, and irisin) that are involved in the muscle repair. The discovery of plasmatic myokines involved in the muscle damage and repair may help to elucidate the endurance exercise muscle adaptations and may yield potential molecular therapeutic targets to treat the myopathies that involve mitochondrial dysfunctions.

## Data Availability Statement

The raw data supporting the conclusions of this article will be made available by the authors, without undue reservation.

## Ethics Statement

The studies involving human participants were reviewed and approved by Ethics Committee of Dante Pazzanese Institute of Cardiology, Brazil (Permit Number: 979/2010). The patients/participants provided their written informed consent to participate in this study.

## Author Contributions

CS carried out the data collection, acquisition analysis, and data interpretation. AS carried out the data collection, participated in its design, and helped draft the manuscript. BM performed acquisition analysis and data interpretation and helped draft the manuscript. JM and RM were responsible for the data collection. HB and HS participated in the experimental design, acquisition analysis, and data interpretation, as well as helped to draft the manuscript. MC-B conceived the study, participated in its design and coordination, helped to perform the statistical analysis, and drafted the manuscript. All authors have read, revised, and approved the final version of the manuscript and agree with the order of presentation of the authors.

## Funding

This work was supported by the Fundação de Amparo à Pesquisa do Estado de São Paulo (FAPESP) [grant number 2018/26269-0].

## Conflict of Interest

The authors declare that the research was conducted in the absence of any commercial or financial relationships that could be construed as a potential conflict of interest.

## Publisher's Note

All claims expressed in this article are solely those of the authors and do not necessarily represent those of their affiliated organizations, or those of the publisher, the editors and the reviewers. Any product that may be evaluated in this article, or claim that may be made by its manufacturer, is not guaranteed or endorsed by the publisher.
